# Shen-Shuai-Ling Formulation Attenuates Renal Interstitial Fibrosis in Chronic Kidney Disease by Regulating SHH-Gli1 Signaling Pathway

**DOI:** 10.1155/2022/3754985

**Published:** 2022-02-12

**Authors:** Ying Li, Haitao Tu, Yan Luo, Weijian Xiong, Hong Liu, Yanying Xiong, Qin Zhang, Huihui Li, Xuan Gao

**Affiliations:** ^1^Department of Nephrology, Chongqing Hospital of Traditional Chinese Medicine, Chongqing 400021, China; ^2^Department of Nephrology, The First Clinical Hospital of Guangzhou Medical University, Guangzhou 510405, China

## Abstract

**Background:**

Shen-Shuai-Ling Formulation (SSLF) has apparent effects on improving renal function, delaying the progression of chronic kidney disease (CKD).

**Methods:**

Fifty male SD rats were randomly divided into 5 groups: Sham group, Model group, SSLF group, CPN group, and C + S group. The morphological changes and the collagen fibers of the rat kidneys were observed by HE staining. The expression of *α*-SMA, Col I, SHH, Gli1, and snail1 was detected by Western blot and qPCR. Then, the cells were divided into the control group, SHH group, and SHH + SSLF serum group.

**Results:**

Compared with the Model group, the fibrosis in SSLF, CPN, and C + S groups was significantly alleviated. And, compared with those in the Model group, the expression of *α*-SMA, Col I, SHH, Gli1, Snail in SSLF, CPN, and C + S groups decreased remarkably.

**Conclusions:**

SSLF remarkably improves renal function and alleviates renal interstitial fibrosis in UUO rats.

## 1. Introduction

CKD has become a prominent public health issue in the world. The global prevalence of CKD in 2017 was 9.1% (697.5 million cases). Nearly one-third of CKD cases occurred in China (132.3 million) or India (115.11 million), with more than 10 million cases in 10 countries and more than 1 million cases in 79 countries. In 2017, CKD caused 1.2 million deaths and was the 12th leading cause of death in the world [1, 2]. The prevention and treatment of CKD and its related diseases pose an immense challenge to the global health system. The main pathological changes of renal interstitial fibrosis (RIF) include myofibroblasts activation, abnormal deposition of the extracellular matrix, and progressive increase of fibrous scars [3, 4]. RIF is a common route and pathological feature in the development of all kinds of CKD leading to end-stage kidney disease. It has been confirmed that the severity of RIF is closely linked to the degree of renal function declination. It is an effective indicator of the disease prognosis. Therefore, it is of great significance to study the mechanism of RIF in order to delay the pathological process of CKD and preserve renal function.

In recent years, a large number of studies have proved that SHH (Sonic hedgehog) signaling pathway is involved in the progress of liver, lung, and bile duct fibrosis [5–8]. Thus, the SHH signaling pathway has become a focus in tissue fibrosis research. Studies have shown that SHH is highly expressed in damaged renal tubular epithelial cells and mainly functions on proliferation and activation of interstitial fibroblasts [9–12]. Many domestic and international experiments have confirmed that the inhibition of the SHH signaling pathway could significantly reduce renal fibrosis and protect renal function [13]. Inhibition of Gli1, a marker protein in the SHH-Gli1 signaling pathway, is the emphasis of research. The expression of Gli1 is a reliable indicator of the activation of the SHH signaling pathway. Scholars abroad have also found that activation of SHH can be precisely controlled by regulating the expression of Snail. Therefore, it is particularly important to seek a treatment scheme to block the activation of the SHH signaling pathway.

SSLF is a prescription developed by Xin Zheng, a master of Traditional Chinese Medicine with more than 60 years of clinical experience [14]. SSLF contains rhubarb, keel, oyster, Dangshen, astragalus, fungus Ganoderma lucidum, Epimedium, safflower, angelica, salvia miltiorrhiza, and dandelion [15]. SSLF has apparent effects on improving renal function, delaying the progression of CKD, and can significantly relieve clinical symptoms such as constipation and abdominal distension. To study SSLF's functional mechanism and understand its potential inhibitory role in renal fibrosis, this study utilizes the UUO model to mimic renal interstitial fibrosis in rats and administers SSLF and/or other medication to them. The pathological changes and cell proliferation in renal fibrosis and the effects of SSLF on the expression of SHH, Gli1, and snail1 protein have been analyzed. The relationship between SSLF and the SHH signaling pathway and the underlying mechanism of SSLF's inhibitory function against RIF is discussed in our study.

## 2. Materials and Methods

### 2.1. Animals and Cells

Fifty clean-grade male SD rats (7 weeks old, (200 ± 20)g) were purchased from the Laboratory Animal Center of Chongqing Institute of Traditional Chinese Medicine (Animal license No. SCXK:20120001). Normal rat kidney interstitial fibroblasts (NRK-49F) were purchased from the American Type Culture Collection (ATCC, Manassas, VA). The animal study was performed according to the National Standards for Laboratory Animals. The animal experiment was approved by the Animal Ethics Committee of Chongqing Hospital of Traditional Chinese Medicine.

### 2.2. Experimental Herbal Formulation

SSLF is composed of rhubarb, *Codonopsis pilosula*, *Astragalus* membranaceus, Os Draconis, Ostreagigastnunb, *Carthamus tinctorius*, Ganoderma lucidum, *Angelica sinensis*, *Salvia miltiorrhiza*, *Epimedium brevicornu* Maxim, and dandelion, etc. SSLF used in our research was processed by the Chongqing Institute of Traditional Chinese Medicine.

### 2.3. Reagents and Instruments

Antibodies used in our experiments included anti-SHH, anti-Gli1, anti-snail1, and anti-GAPDH antibodies (Affinity Biosciences, China). PVDF membrane, BCA Protein Assay Kit, and BeyoECL Moon kit were acquired from Beyotime Biotechnology, China. Cyclopamine was purchased from Selleck Chemicals, USA. Experimental instruments included AU400 automatic biochemical analyzer (OLYMPUS, Japan), BX51T-PHD-J11 microscope (OLYMPUS Company, Japan), image acquisition system CMOS (OLYMPUS Company, Japan), and Image-Pro Plus (Media Cybernetics Company, USA), etc.

### 2.4. Animal Model and Experimental Design

#### 2.4.1. Experimental Animals

The animal experiment was approved by the Animal Ethics Committee of Chongqing Hospital of Traditional Chinese Medicine. Fifty clean-grade male SD rats were randomly divided into five groups with 10 rats in each group: Sham operation (Sham) group, Model group, SSLF group, CPN group, and S + C group. The Sham group were first sedated with 5% sodium pentobarbital. Then, their abdominal cavities were cut open to expose left ureters. Blunt dissection was performed on the left ureters before the closure of abdominal cavities by stratified suture. Besides the Sham group, all the other groups underwent the same surgical procedures to establish UUO model rats: after anesthesia with 5% sodium pentobarbital, the rats' left ureters were exposed and bluntly dissected. Double ligature with 4–0 surgical suture on the upper 1/3 of the ureters was applied to cut off the ureters before closing abdominal cavities. The SSLF group were given 20 mg/kg SSLF intragastrically for 14 days. The dosage was calculated according to Experimental Pharmacology [9]. The rats in the Sham group and Model group were intragastrically given 20 mg/kg of distilled water for 14 days. The CPN group were continuously injected with 10 mg/kg/d of Cyclopamine for 14 days. The S + C group were intragastrically injected with 20 mg/kg/d of SSLF and intraperitoneally injected with 10 mg/kg/d of Cyclopamine for 14 days. After 14 days, tissues and blood were harvested from the rats fasted for 12 hours. SSLF has no obvious side effects [16].

#### 2.4.2. Detection of Blood Biochemical Indexes

The blood samples of rats were taken through the tail vein. The samples were settled at room temperature for one hour and centrifuged at 3500 rpm for 5 minutes. The clear supernatant was used for serum SCr and BUN analysis by an automatic biochemical analyzer.

#### 2.4.3. Histopathological Analysis of the Renal Tissue

The rats' renal tissue was fixed in 4% paraformaldehyde for 24 hours, embedded in paraffin, and sectioned. The 3 *μ*m thick sections were dewaxed, treated with gradient ethanol, stained with Hematoxylin and Eosin (HE), and sealed with neutral resin. The renal tissue was observed by a microscope. The nuclei appeared to be pink or red under the microscope.

#### 2.4.4. Analysis of Renal Tissue Fibrosis

The renal tissue was collected and immersed in a 4% paraformaldehyde tissue fixative. After dehydration, permeation, waxing, and embedding, 2 *μ*m tissue sections were produced with a pathological slicer. PAS (Periodic Acid-Schiff) and Masson staining were performed on the sections. Pathological semiquantitative analysis of glomeruli and tubules was performed using a light microscope. The degree of glomerular sclerosis was determined by selecting 50 glomeruli under 400 times magnification from each slide, and the sclerotic index of the glomeruli was calculated according to the average points.

The severity of sclerotic disease was scored from 1 to 4 points. The mesangial sclerotic area less than 25% was scored as 1 point; the sclerotic area between 25% and 50% was scored as 2 points; the sclerotic area between 50% and 75% was scored as 3 points; the sclerotic area larger than 75% was scored as 4 points. The interstitial score of the renal tubules was analyzed under 200 times magnification; 10 tubules per visual field were observed. According to the range of renal tubular atrophy, tubular type, stromal cell infiltration, and fibrosis, scores of 0 to 3 were assigned: 0 for none, 1 for less than 25%, 2 for 25%–50%, and 3 for 50–75%.

#### 2.4.5. The Expression of Fibrosis-Related Proteins Detected by Western Blot

Renal tissue protein was extracted, quantified, and visualized by Western blot after electrophoresis, membrane transfer, and antibody incubation. Primary antibodies (1∶1000 dilution) for *α*-SMA, Collagen I, SHH, Gli1, and Snail were added for overnight incubation at 4°C on a shaking bed, respectively. Then, the secondary antibody at 1 : 2000 dilution was added, and the membranes were incubated at 37°C for 120 min. After applying ECL color reagent and dark chamber exposure imaging, the gray value of the images was analyzed by Quantity One software. The ratio of the target protein to the GAPDH band absorbance value was calculated to reflect the expression level of the target proteins.

### 2.5. In Vitro Experiments

#### 2.5.1. NRK-49F Cells Stimulated by SHH

NRK-49F cells purchased from ATCC (Manassas, VA) were utilized in the in vitro experiments. The cells were treated with 10% fetal bovine serum, 100000 units/L penicillin, and 100 mg/L streptomycin in the DMEM medium. The activation of NRK-49F cells was induced for 24-, 48-, or 72-hour by 100 ng/L human SHH protein (StemRD Inc., Burlingame, CA). The expression of snail1 and Gli1 in vitro was evaluated by Western blot analysis.

#### 2.5.2. Preparation of SSLF-Containing Serum

Fifty clean-grade male SD rats were given 20 mg/kg of SSLF per day intragastrically. After 7 days, blood was harvested from the rats fasted for 12 hours. The blood samples of rats were settled at room temperature for one hour and centrifuged at 3500 rpm for 5 minutes to obtain SSLF serum stock.

#### 2.5.3. Administration of SSLF In Vitro

A humidified incubator with 5% CO_2_ at 37°C was used to culture the NRK-49F cells. The cells were divided into the following groups: the untreated control group (control); SHH group (SHH) in which cells were induced for 48 hours by 100 ng/L human SHH protein; and SHH + SSLF group (SHH + SSLF) in which cells were treated with 10% SSLF serum, 100000units/L penicillin, and 100 mg/L streptomycin in the DMEM medium and induced for 48 hours by 100 ng/L human SHH protein. An equal amount of nuclear and cytoplasmic extracts was tested for the expression of Gli1, snail1, Collagen I, *α*-SMA, and PCNA by Western blot.

#### 2.5.4. Network Pharmacology

The main targets of the Shenhueling formula were predicted by network pharmacology. The effective active ingredients of SSLF and related targets were searched in TCMSP. Rheumatoid arthritis-related genes were selected according to the GeneCards disease database, and the PPI protein interaction network was constructed to carry out GO enrichment analysis and KEGG pathway enrichment analysis of key targets.

### 2.6. Statistical Analysis

Data analysis was carried out using SPSS 17.0 software. The quantitative data were expressed as mean ± SEM ($\bar{x}$ ± *s*). Independent-samples *t*-test or ANOVA was applied for the statistical analysis. Pairwise comparisons were performed using *t*-tests. *p* value less than 0.05 was considered statistically significant.

## 3. Results

### 3.1. Network Pharmacological Analysis

**Figure 1:**
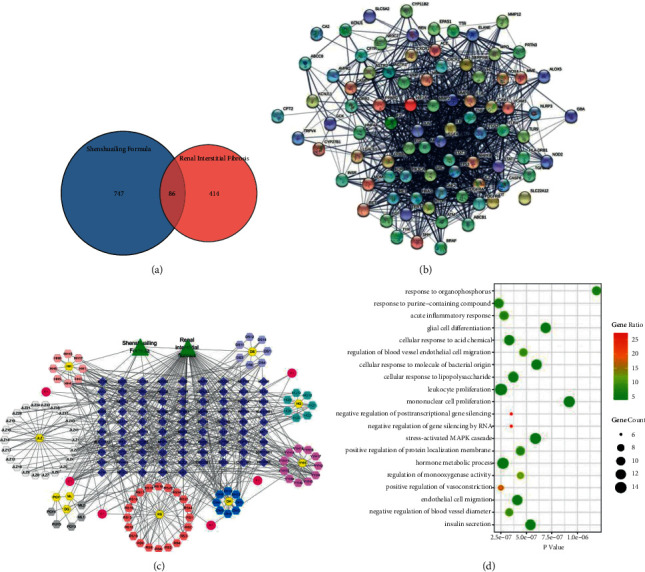
SSLF on renal interstitial fibrosis by network pharmacology analysis. (a) Venn diagrams of intersections of drug and disease targets. (b) String analysis protein interaction network diagram. (c) Drug-active ingredient-target network diagram. (d) Bubble map was analyzed by GO of intersection gene.

We used TCMSP to find 10 major components of SSLF and Swiss TargetPrediction to predict 774 drug target genes. The intersection of drug target genes and 414 targets of renal interstitial fibrosis resulted in a total of 86 targets, which were involved in biological processes and functions such as acute inflammatory response, protein binding, and growth factor receptor binding. The compounds targeting SHH were identified as quercetin, CLR, and epoxyganoderiol A, respectively, which were regarded as the major bioactive compounds (Figure 1).

### 3.2. Animal Experiments

#### 3.2.1. SCr and BUN Levels in Each Group

**Figure 2:**
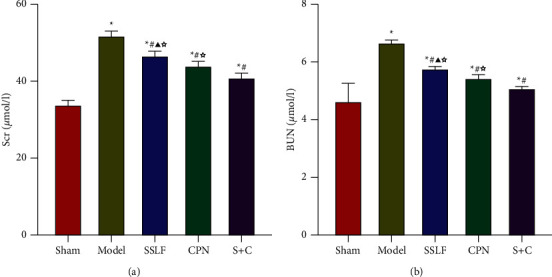
Levels of SCr and BUN in renal tissues in each group. (a) Levels of SCr in renal tissues in each group. (b) Levels of SCr in renal tissues in each group.^*∗*^*p* < 0.05 compared with the Sham group; ^#^*p* < 0.05 compared with the Model group; ^△^*p* ＜ 0.05 compared with the S + C group; ^▲^*p* ＞ 0.05 compared with the CPN group.

Compared with the Sham group, the SCr and BUN levels in all the other groups were significantly higher (*p* < 0.05). In comparison with the Model group, SCr and BUN levels in SSLF, CPN, and S + C groups were significantly lower (*p* < 0.05). In comparison with the S + C group, SCr and BUN in SSLF and CPN groups were significantly higher (*p* < 0.05). There was no significant difference between the SSLF and CPN groups (Figure 2).

#### 3.2.2. Observation of Kidney Samples in Each Group

The kidneys from the Sham group were of normal size, dark red color, and soft to the touch. In the Model group, the left kidneys were obviously swollen and had a dark brown color. The renal capsules were tense; turbid fluid could be seen in the incision. The calyxes of the renal pelvis were dilated and deformed, and the renal cortex appeared to be significantly thinner compared with that of the Sham group. The kidneys in SSLF, CPN, and S + C groups were found to be swollen and dark brown; however, their renal cortex appeared to be slightly thicker than that of the Model group.

#### 3.2.3. Histopathological Changes in Renal Tissues

**Figure 3:**
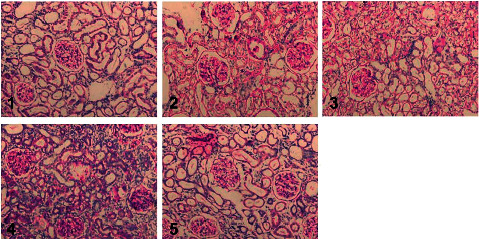
Histopathological changes in each group. 1: Sham group; 2: Model group; 3: SSLF group; 4: CPN group; 5: C + S group (HE staining, ×200).

The HE staining of the Sham group showed normal renal tissue structure. The tubules in renal tissues were arranged neatly, and the basement membrane was continuous. No inflammatory infiltration in the stroma area was found. Compared with the Sham group, the renal tubules in the Model group were obviously dilated and atrophied. Fibrous tissue proliferated in the cortical medulla. The renal interstitial area expanded, and the infiltration of inflammatory cells was apparent. Compared with the Model group, there was less infiltration of renal interstitial inflammatory cells in the SSLF, CPN, and S + C groups. The dilatation and atrophy of renal tubules and fiber proliferation were also alleviated. No significant histopathological differences were seen in the SSLF, CPN, and S + C groups. However, the S + C group appeared to have less pathological changes (Figure 3).

#### 3.2.4. Effects of SSLF on Collagen Fiber Deposition Rate

**Figure 4:**
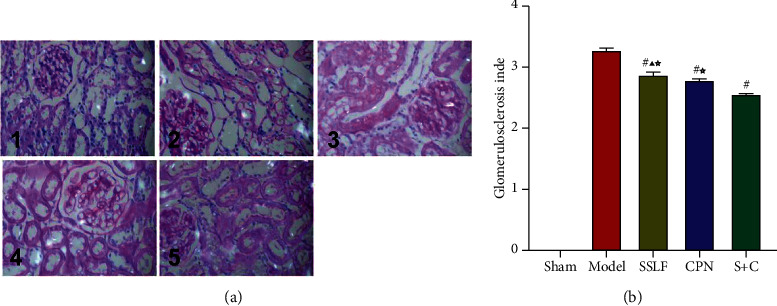
PAS staining to observe renal tissue fibrosis in each group. (a) 1: Sham group. 2: Model group. 3: SSLF group. 4: CPN group. 5: C + S group (PAS staining, ×400). (b) The scores of glomerulosclerosis index in PAS staining.^#^*p* < 0.01 versus Model group, ^☆^*p* < 0.05 versus S + C group, ^▲^*p* ＞ 0.05 versus CPN group.

**Figure 5:**
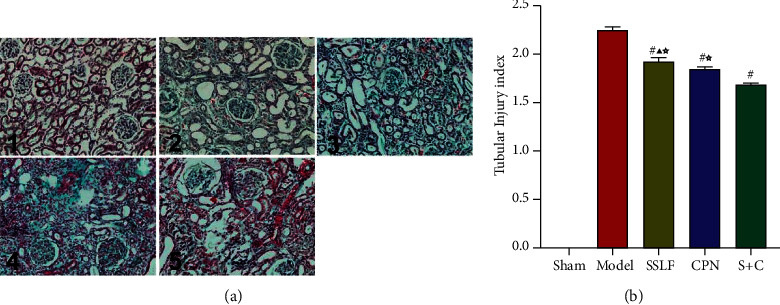
Masson staining to observe renal tissue fibrosis in each group. (a) 1: Sham group; 2: Model group; 3: SSLF group; 4: CPN group; 5: C + S group (Masson staining, ×200). (b) The scores of tubular injury index in Masson staining.^#^*p* < 0.01 compared with the Model group, ^☆^*p* < 0.05 compared with the S + C group, and ^★^*p* > 0.05 compared with the CPN group.

According to the method described in paragraph 1.4.4, the kidney samples from each group were counted and scored. There were no obvious changes in the glomerulus and renal tubule in the Sham group. In the Model group, there were marked changes in the glomerulus, the renal tubule, and the interstitial tissue. The mesangial matrix had increased with hyperplastic mesangial cells, and the wall of the glomerulus was thickened. The capillary was expanded or occluded, and some of the pellets had segmental or spherical sclerosis. The renal tubule was atrophic and exhibited an expanded small tube. A large amount of the protein tube appears, accompanied by widened renal interstitial and infiltration of inflammatory cells. Renal stroma also showed microvascular lesions, with focal distribution, disordered structure, deformation, and reduction of the capillary cavity. The morphological changes in the C + S and CPN groups were slightly improved; the injury scores of glomeruli and tubules were significantly reduced. PAS staining showed that compared with the Model group, the glomerular score in the SSLF group had decreased by 10% (*p* < 0.05), the CPN group had decreased by 12% (*p* < 0.05), and the C + S group had decreased by 26% (*p* < 0.05). There were no significant differences between the SSLF and CPN groups. The renal tubule stroma scores revealed by Masson staining showed that, in comparison with that in the Model group, the score of the SSLF group had decreased by 9% (*p* < 0.05), and that of the C + S group had decreased by 14% (*p* < 0.05). And the glomerular score of C + S group had decreased by nearly 21% (*p* < 0.05). (Figures 4 and 5).

#### 3.2.5. Western Blot and qPCR Analysis

**Figure 6:**
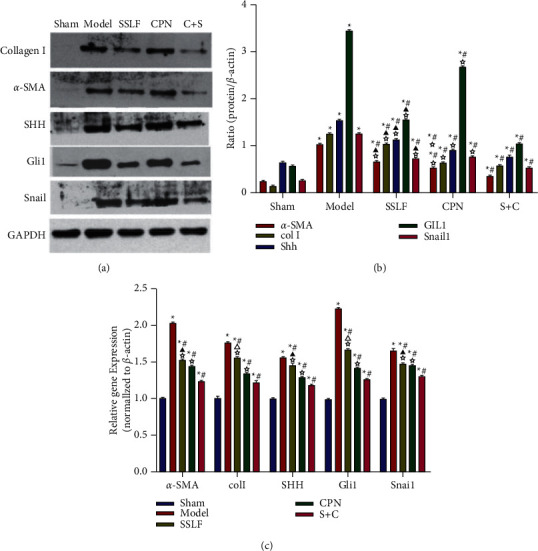
Levels of *α*-SMA, Col I SHH, Gli1, and snail1 in renal tissues in each group. (a) Levels of *α*-SMA, Col I SHH, Gli1, and snail1 were measured by Western blot. (b) Quantitative analysis. (c) Levels of *α*-SMA, Col I SHH, Gli1, and snail1 were measured by qPCR. Data were expressed as mean ± SEM. ^*∗*^*p* < 0.05 compared with the Sham group, ^#^*p* < 0.05 compared with the Model group, ^△^*p* < 0.05 compared with the CPN group, ☆*p* < 0.05 compared with the *S* + *C* group, ^▲^*p* > 0.05 compared with the CPN group, and ^★^*p* > 0.05 compared with the C + S group.

The results of Western blot and qPCR showed the protein expression of *α*-SMA, Collagen I, SHH, Gli1, and snail1 in the renal tissue of the Model group were significantly higher than those of the Sham group (*p* < 0.05). And the protein expressions of *α*-SMA, Collagen I, SHH, Gli1, and snail1 in the renal tissue of SSLF, CPN, and C + S groups were significantly lower than those of the Model group (*p* < 0.05). There was no significant difference in the expression of fibrotic protein markers between CPN and S + C groups (*p* > 0.05). (Figure 6).

### 3.3. In Vitro Experimental Results

#### 3.3.1. NRK-49F Cells Stimulated by SHH

**Figure 7:**
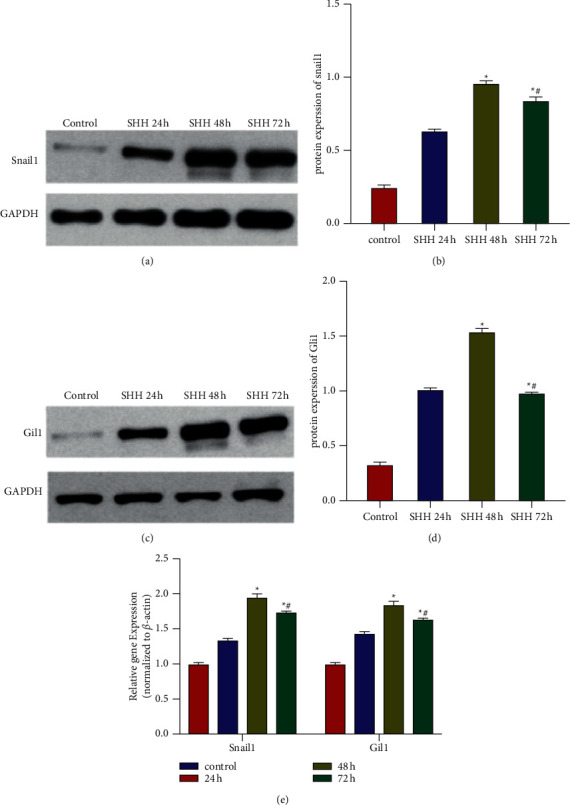
Levels of snail1 and Gli1 in renal tissues in each group. (a, b) Levels of snail1 were measured by Western blot. (c, d) Levels of snail1 were measured by Western blot. (e) Levels of snail1 and Gli1 in renal tissues were measured by qPCR. Data were expressed as mean ± SEM. ^*∗*^*p* < 0.05 compared with the Control group; #*p* < 0.05 compared with the SHH 48 h group.

In comparison with the untreated control, the expressions of snail1 and Gli1 in NRK-49F cells stimulated by SHH (100 ng/ml) were significantly increased. Western blot and qPCR showed that the expression of snail1 and Gli1 in NRK-49F cells had increased in a time-dependent manner upon SHH stimulation. The expression of snail1 and Gli1 peaked at 48 h; thus, we set 48 h as the time point for follow-up experiments (Figure 7).

#### 3.3.2. Effects of SSLF In Vitro

**Figure 8:**
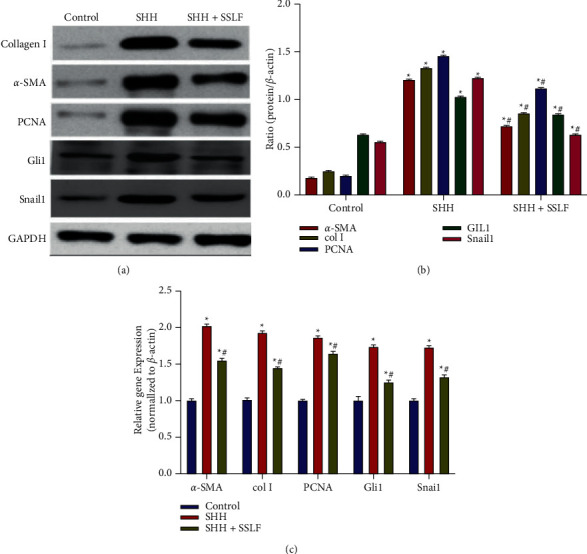
Levels of Snail1 and Gli1 in renal tissues in each group. (a) Levels of collagen, *α*-SMA, PCNA, Gli1, and snail1 were measured by Western blot. (b) Quantitative analysis. (c) Levels of collagenI, *α*-SMA, PCNA, Gli1, and snail1 were measured by qPCR. Data were expressed as mean ± SEM. ^*∗*^*p* < 0.05 compared with the control group; #*p* < 0.05 compared with the SHH 48 h group.

The expressions of Gli1, snail1, Collagen I, *α*-SMA, and PCNA in control and SHH and SHH + SSLF groups were detected by Western blot and qPCR at 48h. The expressions of Gli1, snail1, Collagen I, *α*-SMA, and PCNA in the SHH + SSLF group were significantly lower than those in the SHH group (*p* < 0.05). The SHH and SHH + SSLF groups had higher levels of Gli1, snail1, Collagen I, *α*-SMA, and PCNA expression, compared with control (*p* < 0.05). (Figure 8).

## 4. Discussion

Shen-Shuai-Ling Formulation (SSLF) is a prescription from Traditional Chinese Medicine master Zheng Xin who has more than 60 years of experience. Master Zheng is proficient in the four classics of Traditional Chinese Medicine. Integrating classical and modern knowledge, he specializes in using Chinese herbal medicine to treat renal diseases. SSLF protects renal function by purging turbid toxin, tonifying healthy Qi, improving self-immunity, improving renal blood homeostasis, etc. It reflects the Traditional Chinese Medicine theory of strengthening body resistance and eliminating pathogens.

To study the molecular mechanism of SSLF in the treatment of renal interstitial fibrosis, we screened out major bioactive compounds from SSLF and offered a new understanding of the protection mechanism of SSLF against renal interstitial fibrosis by network pharmacology method. We found that SHH is a common target of SSLF and renal interstitial fibrosis and the SHH signaling pathway has become a focus in tissue fibrosis research, so we chose SHH as the target gene and its downstream signaling pathway to study.

In the clinical study, the serum levels of SCr and BUN of the patients treated with SSLF for one course of treatment have significantly decreased. The results of the present study show that serum levels of SCr and BUN in the UUO rat model are significantly higher than those in the Sham group, suggesting that the UUO model has been successfully established. The serum levels of SCr and BUN in the SSLF treated group are significantly reduced, suggesting that SSLF has protective effects on the renal function of the UUO model rats. There is no significant difference in renal function between the CPN and SSLF groups. And the serum levels of SCr and BUN in the S + C group are the lowest, suggesting that SSLF and CPN have a synergistic effect.

Clinically, the cortex of renal tissue is thinned by a Color Doppler ultrasound of chronic kidney disease. In the Model group, the left kidneys look swollen and dark brown. Manifestations such as tight capsule, turbid effusion, dilatation and deformation of renal pelvis and calyces, and the thinner-than-normal renal cortex indicate the success of UUO model establishment. In the SSLF group, the kidneys are also swollen and dark brown. But the renal cortex is slightly thicker than that in the Model group, suggesting that SSLF has alleviated renal tissue damage and slowed down the progression of renal disease. HE staining reveals that the renal tubules in the Model group are obviously dilated, atrophic, and fibroblastic, accompanied by an increased area of renal interstitium and infiltration of inflammatory cells. According to PAS and Masson staining, the Model group have obvious collagen fiber deposition in renal interstitium, their renal tubules are atrophic or dilated, and there are apparent pathological changes such as glomerular sclerosis and microvascular disease. However, these indicators have been significantly alleviated in SSLF, CPN, and C + S groups according to the scoring system. Col I protein in the ECM can be produced in large quantities after myofibroblast activation. Normally, Col I plays a role in repairing damaged tissues, but the persistent inflammatory reaction can cause myofibroblasts to continuously secrete extracellular matrix protein. The expression of *α* -SMA, a marker of myofibroblast activation, increases as the organ fibrosis exacerbates. Excessive extracellular matrix protein deposition eventually evolves into renal fibrosis. In the Model group, the expressions of *α*-SMA and Col I have significantly increased, indicating severe renal inflammation. Upon SSLF and/or CPN intervention, the expression of *α*-SMA and Col I decreases significantly in myofibroblasts, indicating that SSLF and/or CPN can inhibit the excessive secretion of *α*-SMA and Col I. Thus, SSLF and CPN are able to suppress renal pathological changes and tissue fibrosis alone or synergistically.

Renal interstitial fibrosis (RIF) is a common pathway and pathological feature in various chronic kidney diseases leading to end-stage renal disease. In this process, effective nephrons are gradually lost and renal function declines progressively. The study has confirmed that the severity of RIF is closely related to the degree of renal function declination, and it is a reliable index to determine the prognosis. It has been found that the expression of SHH in renal tubular epithelial cells is significantly upregulated in patients with chronic kidney disease (CKD) with different causes. And other studies have confirmed it in various rat renal fibrosis models as well, suggesting that SHH signaling pathway activation is a common pathological outcome in many kidney diseases [17]. In classical activation of the SHH signaling pathway, SHH binds to the membrane receptor transmembrane protein patched (PTCH) after automatic catalytic cleavage, which relieves the inhibition of smoothed (SMO). SMO activates Gli1 phosphorylation, and then Gli1 enters the nucleus in full-length to initiate the transcription of the target gene [18, 19]. Gli1 is an important multifunctional transcription factor in the SHH signaling pathway, and its activation is a reliable indicator of SHH signaling activity. The SHH-Gli1 pathway is involved in the development of RIF by promoting cell proliferation [20, 21]. Through the study of Gli1-lacZ knockout rats, it has been revealed that the SHH-Gli1 pathway is essential in renal fibrosis [22]. A recent study has found that the SHH-Gli1 pathway can induce the expression of transcription factor snail1 [23]. Further study has found that activation of SHH-Gli1 can sophistically upregulate the expression of *α*-SMA and Collagen III via snail1 in RIF [24,25]. Therefore, it is suggested that the SHH-Gli1-snail1 signaling pathway plays an important role in RIF. In the present study, SSLF and/or CPN inhibit the elevated expression of SHH, Gli1, and snail1 in UUO model rats. We can preliminarily speculate that SSLF and CPN downregulate the SHH-Gli1-snail1 signaling pathway.

Based on the results of in vivo experiments, NRK-49F cells are stimulated with SHH (100 ng/ml). The Gli1 and snail1 expressions in the cells have increased and peaked at 48h, indicating the activation of the SHH-Gli1-snail1 signaling pathway upon SHH stimulation. When SSLF is applied to the cells, the expressions of Gli1 and snail1 are significantly inhibited at 48h. Moreover, the expressions of *α*-SMA, Col I, and PCNA exhibit apparent reduction. Thus, SSLF can significantly inhibit the expression of fibrotic proteins. These results suggest that SSLF inhibits the activation of the SHH-Gli1-snail1 signaling pathway and the proliferation of fibrotic cells, which may be the mechanism of its protective effect against renal fibrosis.

## 5. Conclusion

SSLF can remarkably improve renal function and alleviate renal interstitial fibrosis both in vivo and in vitro. And the underlying mechanism may be related to the inhibition of the SHH-Gli1-Snail signaling pathway.

## Data Availability

The data used to support the findings of this study are available from the corresponding author upon request.
